# Editorial: Underlying neural mechanisms of non-invasion brain stimulation in the treatment of psychiatric disorders: evidence from neuroimaging studies, volume II

**DOI:** 10.3389/fpsyt.2026.1952112

**Published:** 2026-08-11

**Authors:** Yuanjun Xie, Magdalena Sowa-Kućma

**Affiliations:** 1 School of Medical Psychology, Fourth Military Medical University, Xi’an, China; 2 College of Medical Sciences, University of Rzeszow, Rzeszow, Poland

**Keywords:** brain function and structure, neural plasticity, neuroimaging, non-invasive brain stimulation, psychiatric disorders

## Introduction

Non-invasive brain stimulation (NIBS) comprises a heterogeneous family of techniques, including repetitive transcranial magnetic stimulation (rTMS), transcranial direct current stimulation (tDCS), transcranial alternating current stimulation (tACS), and transcutaneous auricular vagus nerve stimulation (taVNS). These approaches differ in their physical principles, spatial and temporal resolution, depth of stimulation, and proposed mechanisms of action, yet all can modulate neural activity without neurosurgical intervention. Over recent decades, NIBS has evolved from an experimental probe of cortical excitability into an established or emerging therapeutic modality for psychiatric disorders. This transition was initially driven by multisite trials establishing the antidepressant efficacy of rTMS (1), and subsequently reinforced by large comparative trials and evidence-based guidelines that refined stimulation parameters, treatment schedules, and clinical indications (2, 3). Nevertheless, the strength of clinical evidence remains uneven across stimulation modalities and diagnostic categories, underscoring the necessity to clarify not only whether NIBS is effective, but also how, where, and in whom it confers therapeutic benefit.

The physiological effects of NIBS extend beyond local changes in cortical excitability beneath the stimulation site. Stimulation-induced activity can propagate through structurally and functionally connected pathways, producing distributed modulations on neural oscillations, cerebral perfusion, functional integration, and large-scale network dynamics (4, 5). Such a distributed mode of action makes neuroimaging particularly valuable for characterizing how NIBS engages targeted circuits and induces changes across connected brain systems. Structural magnetic resonance imaging (MRI) and diffusion imaging characterize individual anatomy and the white-matter pathways that constrain stimulation propagation, whereas functional MRI and arterial spin labeling quantify local and remote changes in activity, connectivity, and perfusion. Electroencephalography and near-infrared spectroscopy provide complementary temporal resolution on electrophysiological and hemodynamic responses. Neuroimaging can therefore serve key translation functions by identifying candidate treatment circuits, guiding individualized targets, verifying target engagement, characterizing treatment-induced plasticity, and delineating predictive biomarkers of clinical response. Connectivity-based targeting studies in major depression illustrate this progression by showing that therapeutic efficacy depends on the functional alignment between accessible cortical targets and deeper symptom-relevant regions, as opposed to standardized superficial coordinates alone (6, 7).

More broadly, these advances align closely with the emerging paradigm of precision psychiatry, in which neuroimaging, computational modeling, and individualized neuromodulation are increasingly integrated to tailor treatment to each patient’s neurobiological profile. Recent developments in artificial intelligence and machine learning further strengthen this framework by enabling multimodal data integration, automated biomarker discovery, and prediction of individual treatment response. Despite this progress, major challenges remain in the reproducibility of neuroimaging biomarkers, the selection of optimal targets, and the translation of mechanistic insights into routine clinical practice. These unresolved issues motivated the second volume of this Research Topic, which brings together complementary clinical, methodological, and translational studies intended to advance the field.

The articles assembled in this second volume of the Research Topic highlight the clinical and mechanistic application of these neuroimaging-guided principles across psychiatric and neurocognitive spectrums. Collectively, they reflect the ongoing transition from empirically derived stimulation protocols toward biologically informed, network-based, and increasingly individualized neuromodulation strategies. These contributions examine how stimulation interacts with individual brain architecture, how therapeutic effects emerge through distributed network reorganization, and how methodological advances may improve the reproducibility and clinical translation of mechanistic findings. The Research Topic is organized around three interrelated themes encompassing neuroimaging-guided personalization of stimulation targets, network-level mechanisms underlying therapeutic change, and critical appraisal of the evidence base and conceptual foundations governing future neuromodulation.

## Personalizing stimulation with individual neuroimaging

A recurring theme across the Research Topic is the use of patient-specific neuroimaging data to personalize stimulation targets. In a randomized controlled trial protocol, Sun et al. propose combining resting-state fMRI, structural MRI, and arterial spin labeling to determine whether four weeks of taVNS can improve post-stroke insomnia while concurrently modulating prefrontal-thalamic functional connectivity, structural integrity, and cerebral perfusion. By integrating these complementary imaging modalities, the study aims to link clinical improvement with convergent circuit-level reorganization. In schizophrenia, Aytulun et al. report a case in which task-based fMRI was used to localize an individualized target adjacent to Heschl’s gyrus for inhibitory rTMS in a patient with clozapine-resistant auditory verbal hallucinations. Hallucination severity was reduced by approximately one-third, accompanied by increased functional connectivity between the auditory target and a fronto-cingulate-insular network implicated in reality monitoring. Although the single-case design precludes conclusions regarding efficacy, the parallel clinical and connectivity changes demonstrate how task activation and network responses can jointly inform mechanistic targeting.

A comparable logic guided Chang et al., who utilized near-infrared spectroscopy to detect bilateral prefrontal hypofunction in a patient with bipolar depression. This physiological pattern informed the selection of an accelerated bilateral high-frequency rTMS protocol, resulting in substantial and sustained symptom improvement. Quinn et al. extended this individualized approach by directly comparing fMRI-guided accelerated intermittent theta-burst stimulation of the left versus right dorsolateral prefrontal cortex (DLPFC) in late-life depression. Both targets engendered equivalent clinical improvement; notably, right-sided stimulation reduced connectivity between the default-mode and limbic networks. These findings suggest that similar clinical outcomes may arise through distinct circuit-level mechanisms, emphasizing that comparable symptom improvement should not be interpreted as evidence of mechanistic equivalence. Extending targeted neuromodulation to refractory psychosis, Raymond et al. applied lesion-network-mapping-informed high-definition tDCS to the right superior temporal sulcus. An accelerated two-day protocol produced reductions in hallucinations and delusions comparable to those observed with a conventional five-day schedule, while changes in electroencephalographic power covaried with clinical benefit. This study highlights the potential value of combining network-informed targeting, accelerated delivery, and electrophysiological markers of target engagement. Although individualized neuroimaging can help determine where stimulation should be delivered, understanding how stimulation reshapes distributed brain networks is equally important for explaining therapeutic efficacy and optimizing treatment strategies.

## Network-level mechanisms of therapeutic change

Subsequent contributions examine how stimulation-induced plasticity is expressed across large-scale networks and how these changes relate to symptomatic or cognitive improvement. In a comprehensive synthesis, Sun et al. integrate evidence across five interacting domains encompassing anti-inflammatory, autonomic, neurotransmitter, neuroimaging, and gut-brain mechanisms, and propose that taVNS may improve depression by reorganizing coupling among the default-mode, cognitive-control, salience, reward, sensorimotor, and visual networks. Their synthesis further emphasizes that these mechanisms are unlikely to operate independently and that multimodal, multi-omics, and machine-learning approaches will be required to determine which biological changes are mechanistically relevant and clinically predictive. In older adults with mild cognitive impairment, Guo et al. found that participants who reverted to cognitively normal status after ten days of rTMS had milder baseline impairment and better-preserved connectivity within the default-mode, executive-control, and frontoparietal networks than those who remained non-reverters. Improvements in immediate and delayed recall, together with baseline connectivity measures, accurately distinguished individuals who subsequently reverted to normal cognition. These findings suggest that preserved network organization may define a window of greater responsiveness and support further investigation of rTMS earlier in the trajectory of cognitive decline.

At the oscillatory level, Debnath et al. show that 5 Hz tACS over the left DLPFC selectively shortened response times during high-load working-memory conditions and increased post-stimulation theta power. The alignment among the stimulation frequency, electrophysiological response, and load-dependent behavioral effect provides primary evidence that frequency-specific neural entrainment may represent one mechanism through which tACS modulates cognitive function. Complementing these clinical and cognitive investigations, Caiani et al. performed computational electric-field modeling in thirty healthy adults and found that incorporating white-matter anisotropy from diffusion imaging substantially altered predicted field strength, orientation, and focality. Moreover, the spatial extent of the modeled electric field was related to the strength of structural connections involving the stimulated region. These results demonstrate that the biological dose delivered is constrained not only by electrode placement and current intensity, but by the individual microstructural architecture, necessitating anatomically realistic field modeling for interpreting interindividual variation in physiological and clinical responses.

## Charting the evidence base and its foundations

The volume also evaluates the broader evidential and conceptual foundations of NIBS research. A systematic review and meta-analysis of 28 randomized trials by Li et al. found that tDCS produced no significant overall improvement in ADHD symptoms or executive function. Exploratory subgroup analyses, however, suggested that anodal montage targeting the right ventromedial prefrontal cortex might selectively improve inhibitory control and working memory. These findings provide an appropriately cautious counterpoint to favorable results from smaller single-site studies, illustrating how montage-specific effects may coexist with a null overall treatment estimate.

A bibliometric analysis of 5,046 publications from 1999 to 2023 by Yang et al. traces the exponential growth of the field, and identifying default-mode network connectivity, functional imaging biomarkers, and theta-burst protocols as prominent and rapidly developing research fronts. Beyond documenting publication trends, this analysis reflects a field-wide transition from region-based stimulation toward network-informed protocols, biomarker development, and accelerated delivery schedules. Finally, González et al. provide a critical historical review tracing psychosurgery from early lobotomy to contemporary connectomic deep-brain stimulation. They argue that therapeutic targets should increasingly be conceptualized as points of access to distributed fronto-limbic circuits, particularly white-matter convergence zones. Although focused primarily on invasive interventions, this connectomic perspective is directly relevant to NIBS, as both approaches aim at modulating distributed symptom-related networks through anatomically accessible cortical nodes.

## Conclusions and future directions

In summary, these contributions support the view that psychiatric and neurocognitive disorders involve disturbances of circuit-level within interconnected neural systems, amenable to target modulation by cortical or peripheral access points. The Research Topic further demonstrates that neuroimaging is moving beyond its traditional role as diagnostic tool by prospectively informing target selection, quantifying individual biological dosing, verifying target engagement, and track neuroplastic reorganization. At the same time, the studies indicate that comparable symptom outcomes may be mediated by distinct patterns of network reorganization and that nominally identical stimulation protocols may generate different electric fields and circuit effects across individuals. The studies included in this Research Topic also highlight persistent challenges that continue to hinder clinical translation. These include modest sample sizes, heterogeneity in stimulation protocols and neuroimaging methodologies, limited external validation of proposed biomarkers, and limited replication across independent cohorts. Addressing these issues will require greater methodological harmonization, transparent analytical workflows, multicenter collaboration, and prospective validation of candidate biomarkers in diverse clinical populations.

Future research should address the major barriers limiting the clinical translation of precision neuromodulation. Multicenter, sham-controlled trials are needed to test whether individualized functional or structural targeting outperforms standard anatomical approaches while adequately controlling for expectancy and nonspecific effects. Future studies should integrate multimodal neuroimaging, computational electric-field modeling, and longitudinal follow-up within the unified cohorts. Such integration e enable shared and modality-specific mechanisms to be distinguished, separate immediate target engagement from delayed neuroplastic effects, and clarify the temporal relationship between neural changes and clinical improvement. Resolving these distinctions will be critical for transforming precision neuromodulation from a mechanistically informed research framework into a reliable, clinically actionable approach to psychiatric care.

Taken together, the studies presented in this Research Topic reflect the continuing evolution of non-invasive brain stimulation from empirically optimized therapeutic protocols toward biologically informed precision interventions. By integrating advanced neuroimaging, computational neuroscience, and individualized stimulation strategies, the field is moving toward more effective, mechanism-based treatments for psychiatric and neurocognitive disorders. Continued multidisciplinary collaboration among neuroscientists, psychiatrists, biomedical engineers, imaging specialists, and computational scientists will be essential to realizing the full clinical potential of precision neuromodulation.
